# Impact of polystyrene microplastics on *Daphnia magna* mortality and reproduction in relation to food availability

**DOI:** 10.7717/peerj.4601

**Published:** 2018-04-18

**Authors:** Rana Aljaibachi, Amanda Callaghan

**Affiliations:** Ecology and Evolutionary Biology Section, School of Biological Sciences, University of Reading, Reading, United Kingdom

**Keywords:** Microplastics, Eco-toxicology, *Daphnia magna*, Chronic toxicity, *Chlorella vulgaris*, Polystyrene, Life history

## Abstract

Microplastics (MPs) in the environment continue to be a growing area of concern in terms of acute and chronic impacts on aquatic life. Whilst increasing numbers of studies are providing important insights into microparticle behaviour and impacts in the marine environment, a paucity of information exists regarding the freshwater environment. This study focusses on the uptake, retention and the impact of 2 µm polystyrene MPs in the freshwater cladoceran *Daphnia magna* in relation to food intake (algae *Chlorella vulgaris*), with MP size chosen to approximately match the cell size of the algae. *Daphnia* were exposed to varied concentrations of MPs and algae. When exposed to a single concentration of MPs *Daphnia* almost immediately ate them in large quantities. However, the presence of algae, even at low concentrations, had a significant negative impact on MP uptake that was not in proportion to relative availability. As MP concentrations increased, intake did not if algae were present, even at higher concentrations of MPs. This suggests that *Daphnia* are selectively avoiding eating plastics. Adult *Daphnia* exposed to MPs for 21 days showed mortality after seven days of exposure in all treatments compared to the control. However significant differences were all related to algal concentration rather than to MP concentration. This suggests that where ample food is present, MPs have little effect on adults. There was also no impact on their reproduction. The neonate toxicity test confirmed previous results that mortality and reproduction was linked to availability of food rather than MP concentrations. This would make sense in light of our suggestion that *Daphnia* are selectively avoiding eating microplastics.

## Introduction

Plastics are used extensively worldwide since they are cheap, easy to manufacture and have properties that allow them to replace natural products, including wood, stone and glass (Cole et al., 2011). Around 90% of the world’s plastics are low- or high-density polyethylene (PE), polypropylene (PP), polyvinyl chloride (PVC), polystyrene (PS) or polyethylene terephthalate (PET) (Andrady & Neal, 2009). Few of these are released into the environment without some form of modification (additives) that can potentially leach into the water and many have properties that allow the adsorption of hydrophobic pollutants, so continuing their persistence and spread throughout the water (Andrady & Neal, 2009). Enormous amounts of plastic enter aquatic environments from the land, with an estimated 4.8–12.7 million tons of plastic entering oceans per year (Jambeck et al., 2015). Visible, larger plastic fragments pose well known risks to marine life and environments, but there is an increasing awareness of the impact of microplastics (MPs) which are defined as plastics less than 5 mm in size (Moore, 2008; Cole et al., 2011; Wright, Thompson & Galloway, 2013). MPs can be generated from the degradation of larger pieces but many are manufactured specifically, for example, for use in cosmetics products such as facial scrubs (Napper et al., 2015). Ingestion of MPs has been demonstrated for many marine organisms and there is considerable evidence to suggest that they are transferred up between different trophic levels (Eriksson & Burton, 2003). MPs in freshwater ecosystems (Besseling et al., 2017) are less studied and yet an increasingly important environmental issue (Wagner, Engwall & Hollert, 2014; Dris et al., 2015). MPs enter freshwater bodies through land-based sources or wastewater treatment plants in addition to the potential degradation of large plastic particles (Mason et al., 2016). Cosmetic products such as facial scrubs, toothpaste or body wash are a primary source of MPs with up to 100,000 MPs released into wastewater in a single use (Napper et al., 2015). Very recently, environmental lobbying in the UK has resulted in a ban of MPs in personal beauty products which will come into force in 2018 as well as a greater awareness of the issues. However, this does not extend to other sources of microplastic (MP) pollution. These include synthetic fibres in clothing (e.g., fleece) following machine washing (Fossi et al., 2014) as well as resin pellets used in plastics manufacture. A survey of the River Thames (UK) shoreline revealed 1–4 mm MPs at concentrations of 22–297 particles/L (Horton et al., 2016). The most dominant MPs were fibres, but in one site downstream of a storm drain, receiving urban runoff, many of the plastics were derived from thermoplastic road-surface marking paints (Horton et al., 2016). In the US effluent from wastewater treatment plants was shown to contain an average of 0.05 ± 0.024 MP particles/L (Mason et al., 2016) and effluent feeding into lakes generated an average of 0.79 ± 0.88 mg/L to 1.56 ± 1.64 mg/L MPs (Lasee et al., 2017). Likewise, a survey of beach sediments from the subalpine Lake Garda in Italy revealed high concentrations of plastics including polystyrene (45.6%), polyethylene (43.1%) and polypropylene (9.8%) as well particles (9–500 µm) of polyamide and polyvinylchloride (Imhof et al., 2013a).

Recent investigations have argued that aquatic organisms have a limited ability to distinguish between food and MPs (Von Moos, Burkhardt-Holm & Köhler, 2012; Fossi et al., 2014). For example, the copepod *Corvus typicus* could not differentiate between algae and 20.6 µm MPs (Cole et al., 2013). Similar results were found when *Acartia clausi* and *Calanus pacificus nauplii* were exposed to MPs in the presence of food (Cole et al., 2013) These factors have led to increasing concerns regarding MPs and have contributed to a recent increase in studies on the impact of MPs on marine and freshwater environments.

Studies on freshwater invertebrate species including *Lumbriculus variegatus, Daphnia magna, Notodromas monacha, Potamopyrgus antipodarum, and Gammarus pulex,* have confirmed that ingestion of MPs occurs (Imhof et al., 2013b). Ingestion and elimination of polyamide fibres and polystyrene MPs were demonstrated in the freshwater amphipod *Gammarus fossarum*  and impacted on food assimilation (Blarer & Burkhardt-Holm, 2016).

Various studies have begun to look at the impact of MPs on growth and reproduction in the model ecotoxicology organism *Daphnia*. *Daphnia magna* (Cladocera, Crustacea) is a freshwater filter feeder with the ability to uptake and ingest small suspended particles from the water (Ebert, 2005).  *Daphnia* usually feed on algae, although they are capable of feeding on bacteria and can consume particles between 1–70 µm in size (Ebert, 2005). *Daphnia* are said to be unable to distinguish between particles size and quality (DeMott, 1986), which implies a lack of selection and likely ingestion of MPs. Tiny carboxylate polystyrene MPs (20 and 1,000 nm) can cross the *Daphnia* gut epithelium and accumulate in lipid storage droplets (Rosenkranz et al., 2009). Bioaccumulation of these nano-polystyrenes is associated with a negative impact on the growth, mortality and reproduction of *D. magna* (Besseling et al., 2014). MP fibres (ground polyethylene terephthalate) from textiles were toxic to unfed *D. magna* but no mortality occured when animals had been fed (Jemec et al., 2016). Degraded macro plastics were also toxic to *D. magna*, increasing inter-brood period and decreasing reproduction at high concentrations whereas responses to cosmetic MPs found no such effect and effects were restricted to the level of nutrition (Ogonowski et al., 2016). This contrasts with the effect of polyethylene MPs where ingestion of high concentrations of 1 µm MPs led to the immobilisation of *D. magna* (Rehse, Kloas & Zarfl, 2016).

The aim of the present study was to add to the growing body of evidence of the impact of MPs through a thorough investigation of the impact of 2 µm micro-polystyrene on *D. magna* on reproduction, growth and mortality. This size was chosen since it is a similar size to *the* alga *Chlorella vulgaris* which is used to feed *Daphnia* This work was undertaken at a point where few had studied the impact of MPs on *Daphnia* and freshwater ecosystems. We hypothesized that *Daphnia* would be unable to differentiate between MPs and algae and that the uptake of both algae and MPs would be equivalent. Given an inability to distinguish between food and non-food, we predicted that the reduction in food ingested would have a significant impact on the fitness of the *D. magna*. Longer term impacts would also be dependent on excretion of MPs as well as general toxicity. We measured the impact on growth and reproduction using standard ecotoxicology 21 days’ life history tests following OECD guideline 211 (OECD, 2012).

## Materials and Methods

### *Daphnia magna* and *Chlorella vulgaris* culture

*Daphnia magna* were obtained from the Water Research Centre (WRC, Medmenham, UK) and cultured at the University of Reading for more than ten years prior to this experiment. Full details of culturing methods are given in (Hooper et al., 2006). *Daphnia* were maintained in Organization for Economic Co-operation and Development (OECD) reconstituted water (media) and fed yeast and *C. vulgaris* var Viridis following the methods of (Hayashi et al., 2008). New cultures of *Daphnia* were prepared with 15 neonates in 1,200 ml beakers filled with OECD media (the progeny of these neonates are the first brood). Juveniles were removed regularly from the culture and the media was changed once a week. The third brood produced by the original 15 neonates were used for experiments.

### Preparation of MPs

MPs used for uptake and depuration experiments were supplied by Sigma-Aldrich, Dorset, UK, (Lot no. MKBQ9691: batch no. 1001856699) as 2 µm carboxylate-modified polystyrene, fluorescent yellow-green (excitation 470 nm; emission 505 nm), density 1.050g/cm^3^. MPs were stored as a stock suspension (2.5 mg mL^−1^) in distilled water and mixed using a vortex prior to dilutions. MPs used for toxicity tests were also supplied by Sigma-Aldrich, Dorset, UK, (Lot no. BCBN6954V: batch no.78452) as 2 µm non-fluorescent polystyrene microplastics. These were stored as an aqueous suspension of 10% solids.

### Microplastic uptake

Adult (18 days) *D. magna* were placed individually in glass beakers filled with 50 ml media and starved for 24 h. They were then exposed to 1.46 × 10^2^ mg/L 2 µm MPs with and without algae (calculated based on carbon 1.00 × 10^−1^mg/L) of *C. vulgaris.* Four replicates of each treatment were exposed for 15, 30, 60, 120 and 240 min in daylight conditions. at 20 ±2 °C. Following exposure *Daphnia* were washed with deionized water to remove MPs that had adhered to the carapace, dried on tissue and placed in an Eppendorf tube and stored at −20 °C.

### Microplastic depuration

In order to evaluate depuration of MPs, 20 *Daphnia* from both treatments (see previous section) were exposed for one hour then transferred into clean media for 15, 30, 60, 120 and 240 min (replicated four times). *Daphnia* were washed twice with deionized water for one minute before freezing at −20 °C.

Frozen *Daphnia* were placed individually into a 1.5 ml Eppendorf tube filled with 500 µl distilled water and homogenised by crushing the *Daphnia* using a glass Kontes Pellet Pestle (Fisher Sciences, Loughborough, UK) for one minute. A further 500 µl distilled water was added to wash the pestle. A 0.2 ml aliquot of the homogenate was filtered onto a black background nucleopore track-etched membrane (Whatman, Kent, UK) <0.2 µm, by using a glass vacuum filter holder connected to a manual air pump. The membrane was examined under the epi-fluorescent microscope (Zeiss Axioskop) under (20×) to count the fluorescent MPs in each treatment.

### Microplastic visual assessment

Four adult *Daphnia* from each treatment for each experiment were observed under an epi-fluorescent microscope (Carl Zeiss Axioskop, Wetzlar, Germany), at (10×) magnification with the main focus on the gut system. Images were taken through a blue filter (excitation 450–490 nm) to differentiate the MPs from algae which fluoresced under a green filter (excitation 510–560 nm) (Figs. S1–S20).

### Differential microplastic uptake under varying food regime

Adult *D. magna* were exposed individually for 60 min to a different combination of MPs and algae concentrations (Table 1). Each treatment was replicated six times and assigned to a randomized block design (where replicates were randomly situated on the laboratory bench to eliminate the contribution of factors other than treatment as an experimental error). Following exposure, *Daphnia* were washed twice with deionized water for one minute before freezing at −20 °C. MPs were quantified as described earlier.

**Table 1:** Concentrations (mg/L) of MPs and algae added to each treatment to study the uptake of microplastics by *Daphnia magna*.

Treatments	Volume µl	Algae concentration (mg/L)	MPs concentrations (mg/L)
Control MP only	50	0	6.93 × 10^−4^
	100	0	1.39 × 10^−3^
	200	0	2.77 × 10^−3^
	400	0	5.54 × 10^−3^
	600	0	8.31 × 10^−3^
	800	0	1.11 × 10^−2^
Algae = MP	50	5.00 × 10^−2^	6.93 × 10^−4^
	100	1.00 × 10^−1^	1.39 × 10^−3^
	200	2.00 × 10^−1^	2.77 × 10^−3^
	400	4.00 × 10^−1^	5.54 × 10^−3^
	600	6.00 × 10^−1^	8.31 × 10^−3^
	800	8.00 × 10^−1^	1.11 × 10^−2^
Algae > MP	50	5.00 × 10^−2^	1.39 × 10^−3^
	100	1.00 × 10^−1^	1.39 × 10^−3^
	200	2.00 × 10^−1^	1.39 × 10^−3^
	400	4.00 × 10^−1^	1.39 × 10^−3^
	600	6.00 × 10^−1^	1.39 × 10^−3^
	800	8.00 × 10^−1^	1.39 × 10^−3^
MP > Algae	50	1.00 × 10^−1^	6.93 × 10^−4^
	100	1.00 × 10^−1^	1.39 × 10^−3^
	200	1.00 × 10^−1^	2.77 × 10^−3^
	400	1.00 × 10^−1^	5.54 × 10^−3^
	600	1.00 × 10^−1^	8.31 × 10^−3^
	800	1.00 × 10^−1^	1.11 × 10^−2^

### Chronic toxicity tests—adults

*Daphnia* (18 days old from the third brood) were placed individually into glass beakers filled with 50 ml media and exposed to one of eight treatments (replicated five times) (Table 2). Media and concentrations were renewed three times per week. In all treatments, life history traits (survival and reproduction) were monitored for 21 days. Neonates were counted daily and removed. Animals unable to swim after gentle stirring for 15 s were considered dead. The experiment was run under laboratory conditions at 20 ±2 °C, and 16 h light :8 h dark.

**Table 2:** Concentrations (mg/L) of MPs and algae added to each treatment to study chronic toxicity in *Daphnia magna*.

Treatments	Algae concentration (mg/L)	MPs (mg/L)
Control low algae	1.00 × 10^−1^	0
Control high algae	8.00 × 10^−1^	0
Algae = MP (low)	1.00 × 10^−1^	1.39 × 10^−3^
Algae = MP (high)	8.00 × 10^−1^	1.11 × 10^−2^
Algae > MP	8.00 × 10^−1^	1.39 × 10^−3^
MP > Algae	1.00 × 10^−1^	1.11 × 10^−2^

### Chronic toxicity test—neonates

A standard chronic toxicity test was conducted in compliance with OECD guideline 211 (OECD, 2012). Five third brood neonates (<24 h) old were placed in glass beakers, and exposed to different treatments in 50 ml media (Table 2). Media and concentrations were renewed three times a week and life history traits (survival, growth and reproduction) were monitored for 21 days. Body length (the area from the top of the head to the base of the tail spine) was measured under a stereomicroscope every other day. The experiment was run at 20 ± 2 °C, with 16 h light: 8 h dark.

### Statistical methods

Data were checked for normality using the Shapiro–Wilk test, SPSS 21.0 (SPSS Incorp., Chicago, Il, USA). The uptake and depuration experiments were normally distributed data and analysed by ANOVA whereas T-tests were conducted to compare between 15 and 240 min.

For the reproduction experiment the Kolmogorov–Smirnov test showed non-normally distributed data. Data were analysed using the Wald chi-square test, which is based on the linearly independent pairwise comparisons among the estimated marginal means of the offspring. T-tests were used to compare the mean reproduction between treatments with and without MPs.

Growth rate data was normally distributed and analysed by UNIANOVA. A post-hoc pairwise comparison was undertaken with t-tests to measure the effect of MPs on the growth rate.

The mortality tests were normally distributed. Analysis was carried out using Minitab V. 17, general linear model and a probit analysis conducted, and the response curve for concentrations was made using a scatterplot.

## Results

### Uptake of MPs

**Figure 1:**
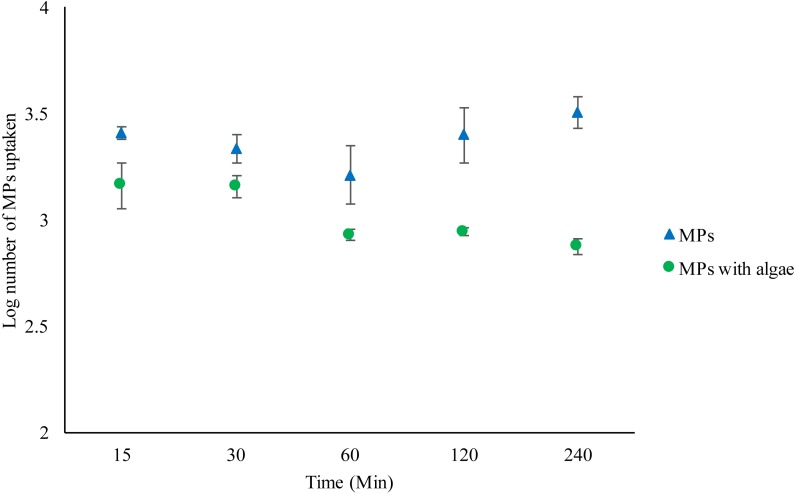
Uptake of 2 µm polystyrene MPs by *Daphnia magna* exposed to MPs only (1.46 ×10^2^ mg/L) or MPs with algae (1.46 ×10^2^ mg/L and 1.00 ×10^−1^ mg/L) over 240 min. Each point represents the mean ± the standard error.

*Daphnia* that were fed with algae plus MPs ate significantly fewer MPs compared to those with just MPs (*F*_1,30_ = 50.702, *P* < 0.001) (Fig. 1) (Tables S1–S2). *Daphnia* treated with MPs and algae not only ate fewer MPs than *Daphnia* without algae, but there was a significant reduction in bead uptake over time (*F*_4,19_ = 5.771, *P* = 0.005). There were significantly fewer MPs in *Daphnia* fed algae after 240 min compared to the number after 15 min’ *t* (d.f. 6) = 2.5, *P* = 0.042. Although *Daphnia* without algae ingested significantly more MPs over time than those fed algae, ingestion over time did not increase significantly (*F*_4,19_ = 1.244, *P* = 0335). The number of MPs ingested after 15 min did not significantly vary from the number of MPs ingested after 240 min *t* (d.f. 6) = − 1.2, *P* = 0.27 (See Figs.  S1–S10).

### Depuration of MPs

**Figure 2:**
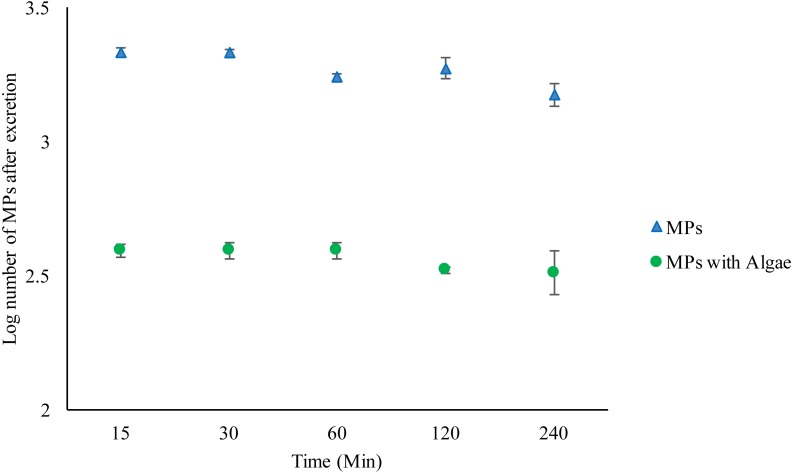
Excretion of 2 µm polystyrene MPs from the gut of *Daphnia magna* exposed to MPs only (1.46 ×10^2^ mg/L) or MPs with algae (1.46 ×10^2^ mg/L and 1.00 ×10^−1^ mg/L)** over 240 min. Each point represents the mean ± the standard error.

As before, *Daphnia* fed algae contained significantly fewer MPs compared to those without algae (*F*_1,30_ = 976.162, *P* < 0.001) (Fig. 2) (Tables S3–S4). Bead excretion in *Daphnia* fed algae did not vary over time (*F*_4,19_ = 1.006, *P* = 0.435). There were no significant differences in bead counts after 240 min compared to the number after 15 min *t* (d.f.6) = 0.978, *P* = 0.366. However, the unfed *Daphnia* excreted a significant number of MPs over time compared to fed *Daphnia* (*F*_4,19_ = 5.452, *P* = 0.006). There were significantly fewer MPs in *Daphnia* after 240 min compared to the number after 15 min *t* (d.f.6) =3.5, *P* = 0.013 (Figs. S11–S20).

### Differential microplastic uptake under varying food regime

**Figure 3:**
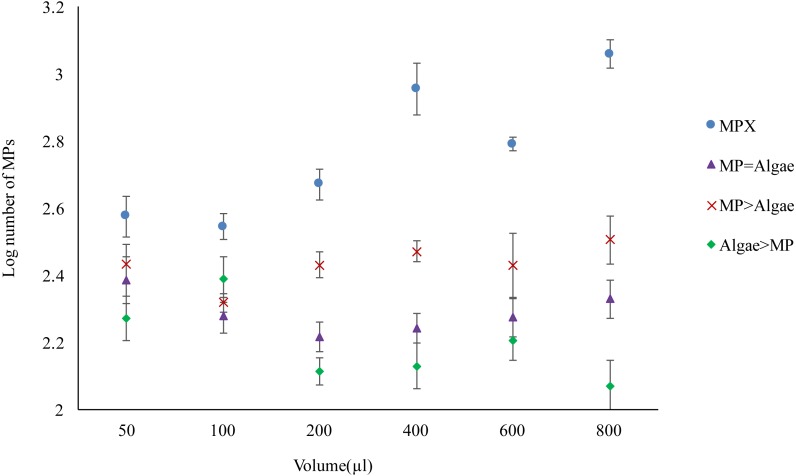
Uptake of 2 µm polystyrene MPs by *Daphnia magna* with and without algae in various volumes (µl) (see Table 1 for actual concentrations). Each point represents the mean ± the standard error.

Four treatment regimes, which varied in the amount of either MPs, algae or both MPs and algae resulted in significant differences in the amount of MPs ingested (*F*_3,120_ = 114.899, *P* < 0.001) (Fig. 3). In unfed *Daphnia* treated with MPs (MPX), the internal bead count increased significantly as the concentration of MPs increased (*F*_5,120_ = 12.849, *P* < 0.001). Where a low amount of food was introduced (MP>Algae) ingested MPs dropped significantly (*F*_3,120_ = 20.788, *p* < 0.001) but did not change with increasing concentrations of MPs (*F*_5,120_ = 1.207, *P* < 0.310). An equal dose of MPs and algae (MP = Algae) was likewise significantly lower than MPX and did not increase as both concentrations increased (*F*_5,120_ = 1.131, *P* < 0.348). Where *Daphnia* were treated with a fixed low concentration of MPs and algal concentrations increased (Algae > MP), the number of MPs decreased significantly (*F*_5,120_ = 4.242, *P* < 0.001).

### Mortality test—adults

**Figure 4:**
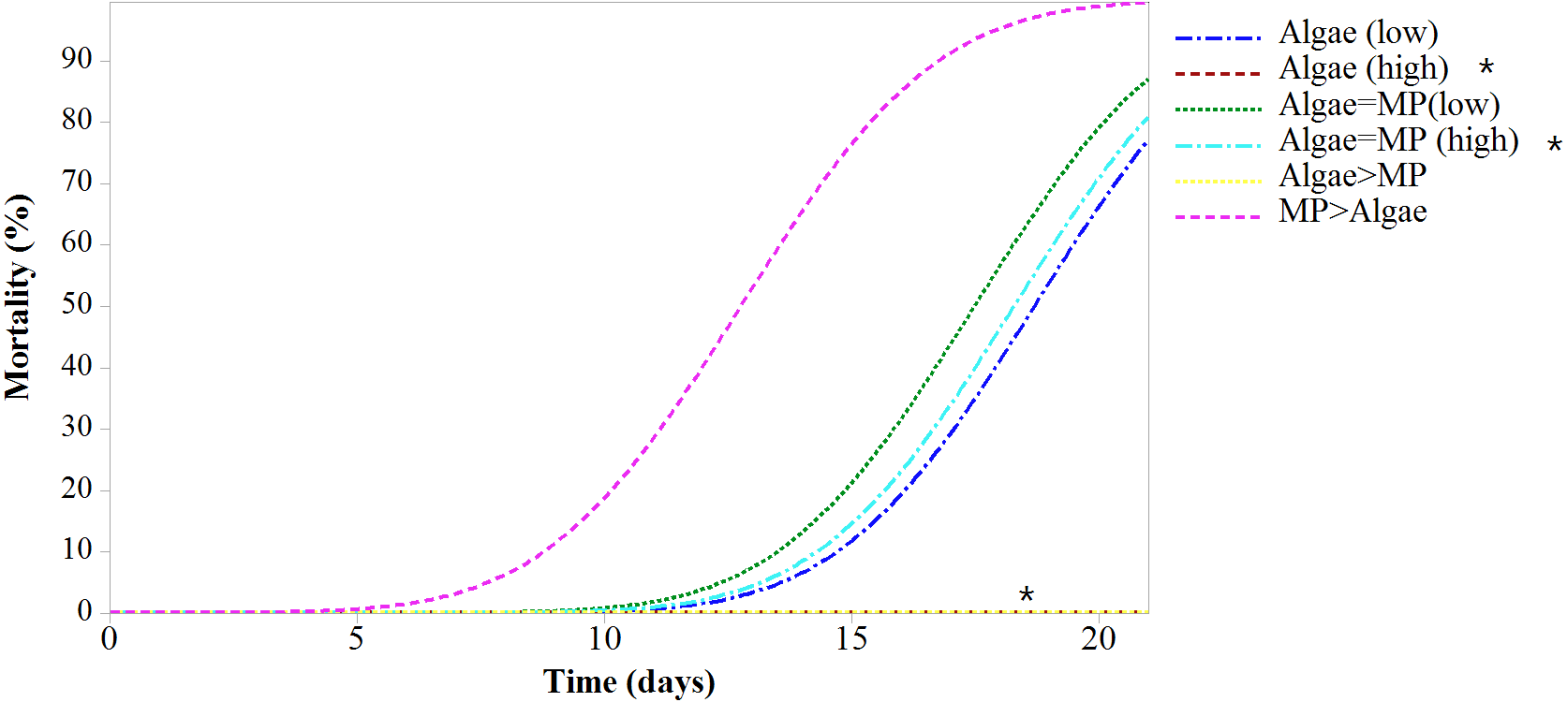
Mortality of *Daphnia magna* expressed as a function of time after chronic exposure to MPs under high and low food conditions for 21 days. Asterisks denote overlap between two treatments.

Mortality rates varied significantly between treatments (*F*_5,618_ = 43.38, *P* < 0.001) (Fig. 4). All treatments had significant mortality compared to the control (algae 8.00 ×10^−1^ mg/L) (*F*_5,618_ = 48.97, *P* < 0.001). A pairwise comparison of mortality in *Daphnia* with restricted food (1.00 ×10^−1^ mg/L algae) plus or minus 1.39 ×10^−3^ mg/L MPs was significant *t* (d.f. 1) = −3.99, *P* < 0.001 and with ample food (8.00 ×10^−1^ mg/L algae) plus or minus 1.11 ×10^−2^ mg/L MPs was highly significant *t* (d.f. 1) = 4.59, *P* < 0.001 since there was no mortality in the treatment without MPs (Table 3).

**Table 3:** Mean ± standard error (S.E.) of lethal time (LT10, LT50 and LT90) of adult *Daphnia magna* exposed to different concentrations (mg/L) of MPs and algae.

Treatments	Concentrations		Lethal time %		
	Algae mg/L	MPs mg/L	LT_10_ ± SE	LT_50_ ± SE	LT_90_ ± SE
Algae (low)	1.00 × 10^−1^	–	7.33 ± 0.825	12.47 ± 0.69	17.6 ± 0.85
Algae (high)	8.00 × 10^−1^	–	39.4 ± 0	44.5 ± 0	49.9 ± 50
Algae = MP (low)	1.00 × 10^−1^	1.39 × 10^−3^	11.22 ± 0.79	16.36 ± 0.69	21.5 ± 0.87
Algae = MP (high)	8.00 × 10^−1^	1.11 × 10^−2^	12.35 ± 0.76	17.48 ± 0.70	22.6 ± 0.92
Algae > MP	8.00 × 10^−1^	1.39 × 10^−3^	39.40 ± 0	44.54 ± 0	49.68 ± 0
MP > Algae	1.00 × 10^−1^	1.11 × 10^−2^	4.95 ± 0.85	10.09 ± 0.70	15.23 ± 0.83

### Reproduction test—adults

**Figure 5:**
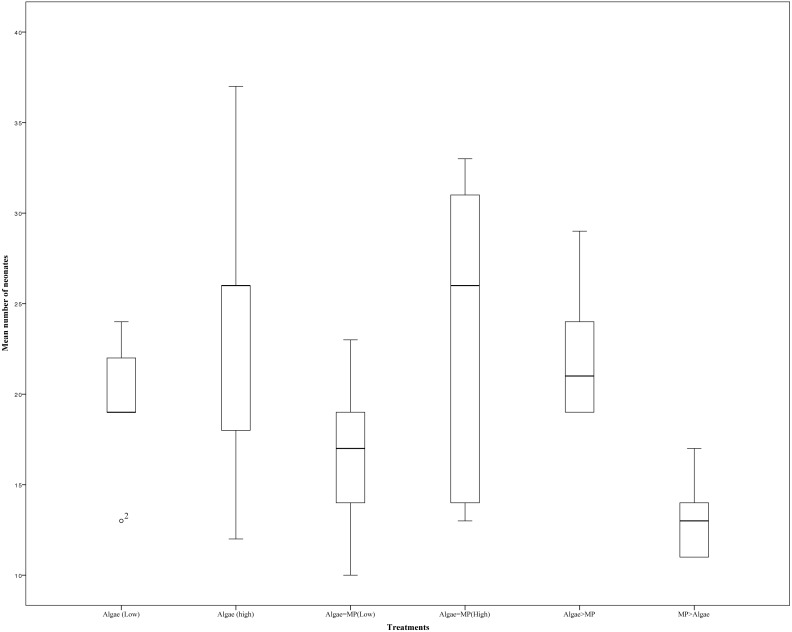
Effects of combinations of high and low MPs and algae concentrations on the mean number of offspring on *Daphnia magna*. Error bars indicate ± 95% confidence intervals and asterisks denote significant differences compared to the control *p* < 0.001.

A 21 day reproduction test on adult *Daphnia* resulted in no significant differences in the mean number of offspring between treatments (Fig. 5) (*X*^2^(5, *N* = 30) = 4.62, *P* = 0.463). There was no significant effect on the mean reproduction between treatments with low algal concentration (1.00 ×10^−1^ mg/L) and those with low algal concentrations with the addition of 1.39 ×10^−3^ mg/L of MPs *t* (d.f. 8) = 0.971, *P* = 0.36. Similarly, there were no significant differences in mean reproduction between *Daphnia* treated with high algal concentrations (8.00 ×10^−1^ mg/L) and those with high algal concentrations with the addition of 1.11 ×10^−2^ mg/L of MPs *t* (d.f. 8) = 0.067, *P* = 0.948.

### Mortality rate—neonate

**Figure 6:**
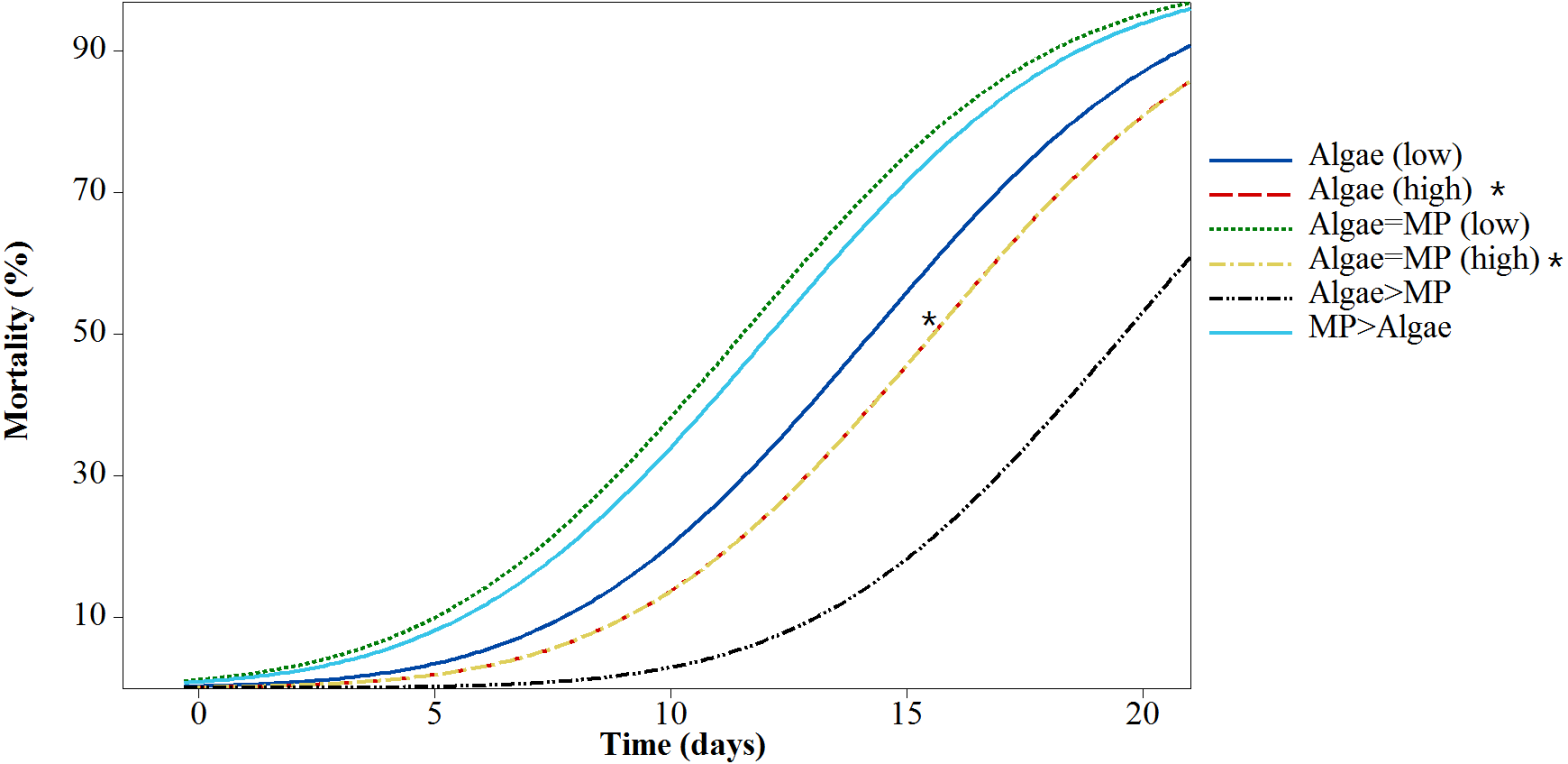
Mortality of neonate *Daphnia magna* after exposure to different treatments of MPs and algae over 21 days. Asterisks denote overlap between two treatments.

In neonates, there was a significant difference in the percentage mortality between treatments (*F*_5,618_ = 26.86, *P* < 0.001) (Fig. 6). The percentage mortality of *Daphnia* fed low algal concentrations and MPs was significantly higher than that of those treated with the same algal concentrations but no MPs *t* (d.f. 5) = 4.24, *P* = <0.001. In contrast, MPs had no impact on mortality when fed to *Daphnia* on a high algal concentration *t* (d.f. 5) = 4.51, *P* = 0.776. Calculated lethal times (LT_10_, LT_50_ and LT_90_) are presented in Table 4.

**Table 4:** Mean ± standard error of lethal time (LT10, LT50 and LT90) of neonate *Daphnia magna* exposed to different concentrations (mg/L) of MPs and algae.

Treatments	Concentrations		Lethal time %		
	Algae mg/L	MPs mg/L	LT10 ± SE	LT50 ± SE	LT90 ± SE
Algae (low)	1.00 × 10^−1^	–	7.71 ± 0.39	14.23 ± 0.35	20.75 ± 0.42
Algae (high)	8.00 × 10^−1^	–	9.02 ± 0.4	15.54 ± 0.36	22.06 ± 0.44
Algae = MP (low)	1.00 × 10^−1^	1.39 × 10^−3^	4.98 ± 0.41	11.51 ± 0.35	18.03 ± 0.41
Algae = MP (high)	8.00 × 10^−1^	1.11 × 10^−2^	9.03 ± 0.4	15.55 ± 0.36	22.07 ± 0.44
Algae > MP	8.00 × 10^−1^	1.39 × 10^−3^	13.07 ± 0.42	19.59 ± 0.42	26.11 ± 0.52
MP > Algae	1.00 × 10^−1^	1.11 × 10^−2^	5.57 ± 0.4	12.09 ± 0.34	18.61 ± 0.4

### Reproduction test—neonate

**Figure 7:**
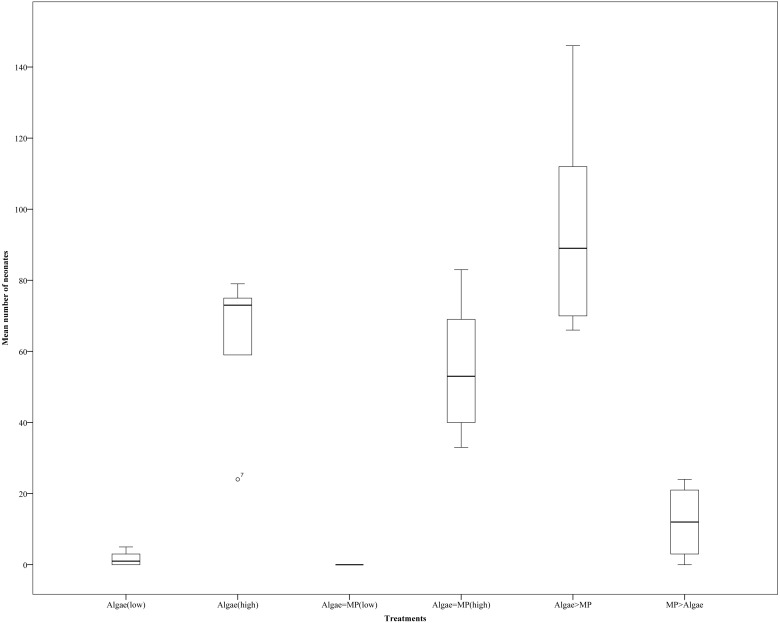
*Daphnia magna* reproduction (neonate production) after 21 days’ exposures to a range of MP and algae treatments (algae (low), algae (high), Algae = MP(low), Algae = MP(high), Algae > MP, MP > Algae). Error bars indicate ± 95% confidence intervals and asterisks denote significant differences compared to the control *p* < 0.001.

The reproduction rate was significantly different between adults that had been treated since they were neonates with high and low algae concentrations (Fig. 7) (*X*^2^(5, *N* = 28) = 618, *P* > 0.001), this was because all treatments that included (8.00 ×10^−1^ mg/L) algae had significantly higher reproduction compared to those on (1.00 ×10^−1^ mg/L). There were no significant differences in reproduction between identical food regimes with and without MPs (low algae concentration (1.00 ×10 ^−1^ mg/L) in the presence of MPs (1.39 ×10 ^−3^ mg/L) *t* (d.f. 7) = 1.63, *p* = 0.146, high algal concentration (8.00 ×10^−1^ mg/L) in addition to MPs (1.11 ×10^−2^ mg/L) *t* (d.f. 8) = 0.46, *P* = 0.652).

### Growth rate

**Figure 8:**
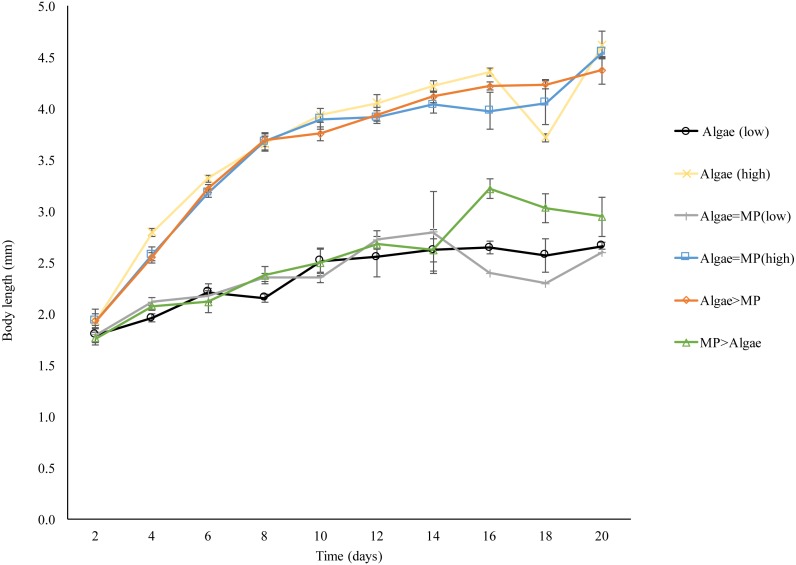
Effect of 21 days’ exposure to different combinations of MPs and algae (Algae (low), algae (high), Algae = MP(low), Algae = MP(high), Algae > MP, MP > Algae) on body length of *Daphnia magna*. Each point represents the mean of five replicates ± standard error.

*Daphnia* body length increased significantly in all treatments over 21 days compared to the initial size (*F*_5,274_ = 166.8, *P* < 0.001) (Fig. 8). *Daphnia* growth was lower in animals exposed to low algae concentrations (1.00 × 10^−1^ mg/L) compared to those given high algae concentrations (8.00 ×10^−1^ mg/L) *t* (d.f. 278) = − 14.5, *P* < 0.001. MPs significantly reduced growth in both algal food regimes (1.00 × 10 ^−1^ mg/L, *F*_2,126_ = 3.009, *P* = 0.05); (8.00 ×10^−1^ mg/L, *F*_2,149_ = 0.63, *P* = 0.05).

## Discussion

Previous studies have suggested that *D. magna* find it hard to distinguish between MP and food particles in the media (Wiedner & Vareschi, 1995). This study was therefore designed to look at the interaction between MP ingestion and food intake, with MP size chosen to approximately match the cell size of the algae. Algae concentrations were chosen based on the minimum and maximum normal daily feeding of *Daphnia* (Hooper et al., 2006), while MPs concentrations were chosen to be close to the algae concentrations volume/volume. When exposed to a single concentration of MPs *Daphnia* almost immediately ate them in large quantities. Previous studies have demonstrated that *Daphnia* will feed on polystyrene, beating their appendages at a constant rate regardless of the food concentration (Pavlaki et al., 2014). The same study also found that plastics of different sizes (1.1 µm and 5.7 µm) were ingested in proportion to their concentration (Pavlaki et al., 2014). This could explain the fact that the amount eaten was reduced when food (algae) was present, which suggests a simple competition for uptake. This hypothesis was further tested by mixing concentrations of algae and MPs. Here there was a direct correlation between MP uptake and concentration so that *Daphnia* ate more MPs as more were available. However, the presence of algae, even at low concentrations, had a significant negative impact on MP uptake. Although MP concentrations increased, intake did not if algae were present, even at higher concentrations of MPs. A high concentration of algae significantly reduced the uptake of a low concentration of MPs so that they were not ingested in direct proportion. Therefore, unlike the study looking at the uptake of different sized polystyrene, there is no evidence that MPs are eaten in proportion to their concentration when algae are present. This implies selectivity (Pavlaki et al., 2014). Previous studies have demonstrated that *Daphnia* are not blindly filtering particles from the water, since particles smaller than the mesh size of their appendages are still eaten (Pavlaki et al., 2014). The uptake of particles is strongly influenced by surface chemistry (negative charges increase uptake) and wettability (which can be reduced by the presence of surfactants to decrease uptake) (Pavlaki et al., 2014). There is also some evidence that *Daphnia* can discriminate artificial from natural particles by taste for instance selecting to feed on phytoplankton rather than clay particles (DeMott, 1986). A previous study noted that MPs tended to form aggregates with algae, thereby effectively increasing the food particle size, with a reduction in MP ingestion when algae were present (Long et al., 2015). There was no evidence of aggregation in any of the experiments presented here pre-feeding.

Where no food was offered, the number of MPs in the *Daphnia* gut remained relatively stable with no significant difference between the number in the gut at the beginning and the end of the experiment. However, there was a significant reduction in MPs over time when food was present.

The excretion of particles was further investigated by measuring the depuration of MPs following a 1 h exposure to MPs followed by clean OECD water for 15, 30, 60, 120 and 240 min. The results showed that the number of MPs in the *Daphnia* decreased over time, suggesting excretion. This has been shown before, with small MPs clearing more rapidly than larger ones (Rosenkranz et al., 2009). However, in the individuals exposed to MPs and food particles, the number of MPs in the gut hardly decreased over time. It is not clear why this is the case and it could be that MPs and food aggregate in the gut, making it hard to excrete MPs. Additionally, it could be that *Daphnia* is re-ingesting the plastic particles after excretion into the media.

*Daphnia* exposed to MPs for 21 days showed mortality after seven days of exposure in all treatments compared to the controls. However, treatments with high MP concentrations and low food levels had the highest mortality compared to the control with low food concentrations. Treatments with high MPs and ample food showed lower mortality compared to the control with low food concentrations. This suggests that where ample food is present, MPs have little effect on adults.

There was also no impact on their reproduction. Again, food levels were more important than MPs concentrations. Similar results had been found recently by Ogonowski et al. (2016), when exposed *Daphnia* to primary MPs or kaolin with low and high food concentrations, results showed life history traits more related to food concentration rather than MPs.

The neonate toxicity test confirmed previous results that mortality was linked to availability of food rather than MP concentrations. *Daphnia* exposed to high food concentrations show higher survival despite MP treatment and reproduction was dramatically decreased in treatments with low food concentrations. Only in the treatment with low food concentrations and low doses of MPs could we see a potential impact of MP with no reproduction. This issue had been investigated previously by exposing the *Daphnia* to metal and different food concentrations which shows that chronic toxicity is linked more to food availability rather than metals; however, that metal get more toxic when there is low food level (Pavlaki et al., 2014).

Growth rate was more effected by food concentrations rather that MPs, *Daphnia* treated with high food concentrations grow are twice as large as those with low food availability. Similar results were obtained elsewhere with both *Daphnia* and marine isopods *Idotea emarginata* (Hamer et al., 2014; Ogonowski et al., 2016). This is in contrast to work published on the effect of ∼70 nm nano-polystyrenes (Nano-PS) on both *Daphnia* and algae (*Scenedesmus obliquus)* (Besseling et al., 2014). Nano-PS negatively impacted population growth and reduced chlorophyll concentrations in the algae. *Daphnia* exposed to Nano-PS had reduced body sizes and the numbers and body size of neonates were lower. Nano-PS also caused high numbers of neonate malformations. However the difference here, apart from the size, is that the authors didn’t use pristine polystyrene but aged the nano-PS with the algae for 5 days (Besseling et al., 2014). Their comparison between aged and pristine nano-PS demonstrated that pristine MPs may not represent the full impact of the exposure.

## Conclusions

Our research was designed to determine the effect of 2 µm MPs on *Daphnia magna* in the presence of algae *Chlorella vulgaris.* This was an experimental approach and was not intended to reflect environmental concentrations of MPs. There is no accurate measurement of 2 µm MPs in freshwater environment and this particular size of plastic can be generated from either a primary source such as cosmetics, or from the degradation of large plastic particles (Connors, Dyer & Belanger, 2017).

The uptake of MPs decreased in the presence of algae and excretion of MPs reduced. The concentration of MPs ingested did not increase with concentration when algae were available which indicates that the *Daphnia* is selectively eating the algae rather than MPs. Chronic toxicity tests (mortality and reproduction rate) found no toxic effect after a 96 h of exposure although seven days of exposure to high concentration of MPs increased mortality. Life history traits of neonates (mortality, reproduction and growth rate) was mainly linked to food concentrations rather than MPs that could confirm *Daphnia* select the food particles rather than MPs.

The study presented here was undertaken to look at the impact of the MPs themselves and as such our results have been obtained with clean MPs that have not been exposed to any contaminants. Environmental MPs are likely to mix with other contaminants, some of which could bind to them and alter their toxicity. Therefore, a future direction of research should include investigating the toxicity of MPs collected from the aquatic environment or in mixtures with known freshwater pollutants such as pesticides.

##  Supplemental Information

10.7717/peerj.4601/supp-1 Figure S1 After 15 min exposure to MPs Raw data image of *Daphnia magna* after 15 min of exposure to microplastics only observed under an epi-fluorescent microscope (Carl Zeiss Axioskop, Germany), at (10×) magnification with the main focus on the gut system. Images were taken through a blue filter (excitation 450–490 nm).

10.7717/peerj.4601/supp-2 Figure S2 After 30 min exposure to MPs Raw data image of *Daphnia magna* after 30 min of exposure to microplastics only observed under an epi-fluorescent microscope (Carl Zeiss Axioskop, Germany), at (10×) magnification with the main focus on the gut system. Images were taken through a blue filter (excitation 450–490 nm).

10.7717/peerj.4601/supp-3 Figure S3 After 60 min exposure to MPs Raw data image of *Daphnia magna* after 60 min of exposure to microplastics** only observed under an epi-fluorescent microscope (Carl Zeiss Axioskop, Germany), at (10×) magnification with the main focus on the gut system. Images were taken through a blue filter (excitation 450–490 nm).

10.7717/peerj.4601/supp-4 Figure S4 After 120 min exposure to MPs Raw data image of *Daphnia magna* after 120 min of exposure to microplastics** only observed under an epi-fluorescent microscope (Carl Zeiss Axioskop, Germany), at (10×) magnification with the main focus on the gut system. Images were taken through a blue filter (excitation 450–490 nm).

10.7717/peerj.4601/supp-5 Figure S5 After 240 min exposure to MPs Raw data image of *Daphnia magna* after 240 min of exposure to microplastics** only observed under an epi-fluorescent microscope (Carl Zeiss Axioskop, Germany), at (10×) magnification with the main focus on the gut system. Images were taken through a blue filter (excitation 450–490 nm).

10.7717/peerj.4601/supp-6 Figure S6 After 15 min exposure to MPs and algae Raw data image of *Daphnia magna* after 15 min of exposure to microplastics** and algae observed under an epi-fluorescent microscope (Carl Zeiss Axioskop, Germany), at (10×) magnification with the main focus on the gut system. Images were taken through a blue filter (excitation 450–490 nm).

10.7717/peerj.4601/supp-7 Figure S7 After 30 min exposure to MPs and algae Raw data image of *Daphnia magna* after 30 min of exposure to microplastics** and algae observed under an epi-fluorescent microscope (Carl Zeiss Axioskop, Germany), at (10×) magnification with the main focus on the gut system. Images were taken through a blue filter (excitation 450–490 nm).

10.7717/peerj.4601/supp-8 Figure S8 After 60 min exposure to MPs and algae Raw data image of *Daphnia magna* after 60 min of exposure to microplastics** and algae observed under an epi-fluorescent microscope (Carl Zeiss Axioskop, Germany), at (10×) magnification with the main focus on the gut system. Images were taken through a blue filter (excitation 450–490 nm).

10.7717/peerj.4601/supp-9 Figure S9 After 120 min exposure to MPs and algae Raw data image of *Daphnia magna* after 120 min of exposure to microplastics** and algae observed under an epi-fluorescent microscope (Carl Zeiss Axioskop, Germany), at (10×) magnification with the main focus on the gut system. Images were taken through a blue filter (excitation 450–490 nm).

10.7717/peerj.4601/supp-10 Figure S10 After 240 min exposure to MPs and algae Raw data image of *Daphnia magna* after 240 min of exposure to microplastics** and algae observed under an epi-fluorescent microscope (Carl Zeiss Axioskop, Germany), at (10×) magnification with the main focus on the gut system. Images were taken through a blue filter (excitation 450–490 nm).

10.7717/peerj.4601/supp-11 Figure S11 Excretion after 15 min from exposure to MPs only Raw data image of *Daphnia magna* after 15 min excretion of microplastics when exposed to MPs only, observed under an epi-fluorescent microscope (Carl Zeiss Axioskop, Germany), at (10×) magnification with the main focus on the gut system. Images were taken through a blue filter (excitation 450–490 nm).

10.7717/peerj.4601/supp-12 Figure S12 Excretion after 30 min from exposure to MPs only Raw data image of *Daphnia magna* after 30 min excretion of microplastics when exposed to MPs only, observed under an epi-fluorescent microscope (Carl Zeiss Axioskop, Germany), at (10×) magnification with the main focus on the gut system. Images were taken through a blue filter (excitation 450–490 nm).

10.7717/peerj.4601/supp-13 Figure S13 Excretion after 60 min from exposure to MPs only Raw data image of *Daphnia magna* after 60 min excretion of microplastics when exposed to MPs only, observed under an epi-fluorescent microscope (Carl Zeiss Axioskop, Germany), at (10×) magnification with the main focus on the gut system. Images were taken through a blue filter (excitation 450–490 nm).

10.7717/peerj.4601/supp-14 Figure S14 Excretion after 120 min from exposure to MPs only Raw data image of *Daphnia magna* after 120 min excretion of microplastics when exposed to MPs only, observed under an epi-fluorescent microscope (Carl Zeiss Axioskop, Germany), at (10×) magnification with the main focus on the gut system. Images were taken through a blue filter (excitation 450–490 nm).

10.7717/peerj.4601/supp-15 Figure S15 Excretion after 240 min from exposure to MPs only Raw data image of *Daphnia magna* after 240 min excretion of microplastics when exposed to MPs only, observed under an epi-fluorescent microscope (Carl Zeiss Axioskop, Germany), at (10×) magnification with the main focus on the gut system. Images were taken through a blue filter (excitation 450–490 nm).

10.7717/peerj.4601/supp-16 Figure S16 Excretion after 15 min from exposure to MPs and algae Raw data image of *Daphnia magna* after 15 min excretion of microplastics when exposed to MPs and algae, observed under an epi-fluorescent microscope (Carl Zeiss Axioskop, Germany), at (10×) magnification with the main focus on the gut system. Images were taken through a blue filter (excitation 450–490 nm).

10.7717/peerj.4601/supp-17 Figure S17 Excretion after 30 min from exposure to MPs and algae Raw data image of *Daphnia magna* after 30 min excretion of microplastics when exposed to MPs and algae, observed under an epi-fluorescent microscope (Carl Zeiss Axioskop, Germany), at (10×) magnification with the main focus on the gut system. Images were taken through a blue filter (excitation 450–490 nm).

10.7717/peerj.4601/supp-18 Figure S18 Excretion after 60 min from exposure to MPs and algae Raw data image of *Daphnia magna* after 60 min excretion of microplastics when exposed to MPs and algae, observed under an epi-fluorescent microscope (Carl Zeiss Axioskop, Germany), at (10×) magnification with the main focus on the gut system. Images were taken through a blue filter (excitation 450–490 nm).

10.7717/peerj.4601/supp-19 Figure S19 Excretion after 120 min from exposure to MPs and algae Raw data image of *Daphnia magna* after 120 min excretion of microplastics when exposed to MPs and algae, observed under an epi-fluorescent microscope (Carl Zeiss Axioskop, Germany), at (10×) magnification with the main focus on the gut system. Images were taken through a blue filter (excitation 450–490 nm).

10.7717/peerj.4601/supp-20 Figure S20 Excretion after 240 min from exposure to MPs and algae Raw data image of *Daphnia magna* after 240 min excretion of microplastics when exposed to MPs and algae, observed under an epi-fluorescent microscope (Carl Zeiss Axioskop, Germany), at (10×) magnification with the main focus on the gut system. Images were taken through a blue filter (excitation 450–490 nm).

10.7717/peerj.4601/supp-21 Table S1 Average number of MPs uptake in treatments exposed to MPs only ±standard error (S.E.)

10.7717/peerj.4601/supp-22 Table S2 Average number of MPs uptake in treatments exposed to MPs and algae ±standard error (S.E.)

10.7717/peerj.4601/supp-23 Table S3 Average number of MPs after excretion in treatments exposed to MPs only ±standard error (S.E.)

10.7717/peerj.4601/supp-24 Table S4 Average number of MPs after excretion in treatments exposed to MPs and algae ±standard error (S.E.)

10.7717/peerj.4601/supp-25 Data S1 Raw data for microplastics count for uptake of microplastics over time, these data applied for data analysis and preparation of Fig. 1

10.7717/peerj.4601/supp-26 Data S2 Raw data for microplastics count after excretion over time, these data applied for data analysis and preparation of Fig. 2

10.7717/peerj.4601/supp-27 Data S3 Raw data for microplastics count for uptake of microplastics through different concentrations of MPs and algae, these data applied for data analysis and preparation of Fig. 3 and Table 1

10.7717/peerj.4601/supp-28 Data S4 Raw data of mortality test for adult *Daphnia magna* over 21 days, these data applied for data analysis and preparation of Fig. 4 and Table 3

10.7717/peerj.4601/supp-29 Data S5 Raw data of reproduction test for adult *Daphnia magna* over 21 days, these data applied for data analysis and preparation of Fig. 5

10.7717/peerj.4601/supp-30 Data S6 Raw data of mortality test for neonates *Daphnia magna* over 21 days, these data applied for data analysis and preparation of Fig. 6 and Table 4

10.7717/peerj.4601/supp-31 Data S7 Raw data of reproduction test for neonates *Daphnia magna* over 21 days, these data applied for data analysis and preparation of Fig. 7

10.7717/peerj.4601/supp-32 Data S8 Raw data of growth rate for *Daphnia magna* over 21 days, these data applied for data analysis and preparation of Fig. 8

## Additional Information and Declarations

### Data Availability

The following information was supplied regarding data availability: The raw data are provided in the Supplemental File.
