# Supplementary material for: Impact of polystyrene microplastics on Daphnia magna mortality and reproduction in relation to food availability
Source: PeerJ. 2018 Apr 18;6:e4601. doi: 10.7717/peerj.4601 (PMC5911131; doi:10.7717/peerj.4601)
Supplement: Figure S17 — Raw data image of Daphnia magna after 30 min excretion of microplastics when exposed to MPs and algae, observed under an epi-fluorescent microscope (Carl Zeiss Axioskop, Germany), at (10×) magnification with the main focus on the gut system. Images were taken through a blue filter (excitation 450–490 nm). [file peerj-06-4601-s017.pdf]

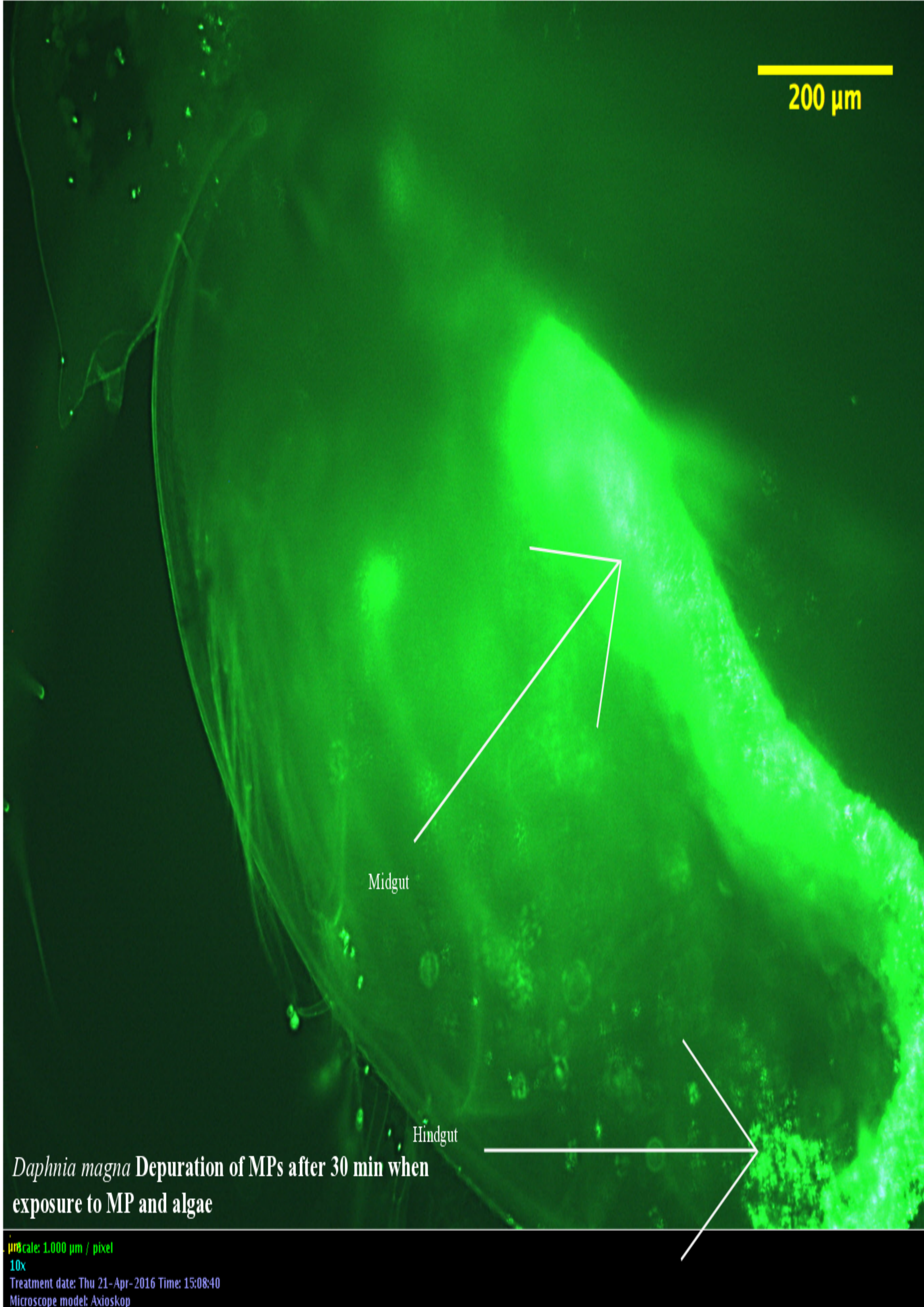
200 µm
Midgut
Hindgut
Daphnia magna Depuration of MPs after 30 min when exposure to MP and algae
µm Scale: 1.000 µm / pixel
10x
Treatment date: Thu 21-Apr-2016 Time: 15:08:40
Microscope model: Axioskop
