# Supplementary material for: Impact of polystyrene microplastics on Daphnia magna mortality and reproduction in relation to food availability
Source: PeerJ. 2018 Apr 18;6:e4601. doi: 10.7717/peerj.4601 (PMC5911131; doi:10.7717/peerj.4601)
Supplement: Figure S19 — Raw data image of Daphnia magna after 120 min excretion of microplastics when exposed to MPs and algae, observed under an epi-fluorescent microscope (Carl Zeiss Axioskop, Germany), at (10×) magnification with the main focus on the gut system. Images were taken through a blue filter (excitation 450–490 nm). [file peerj-06-4601-s019.pdf]

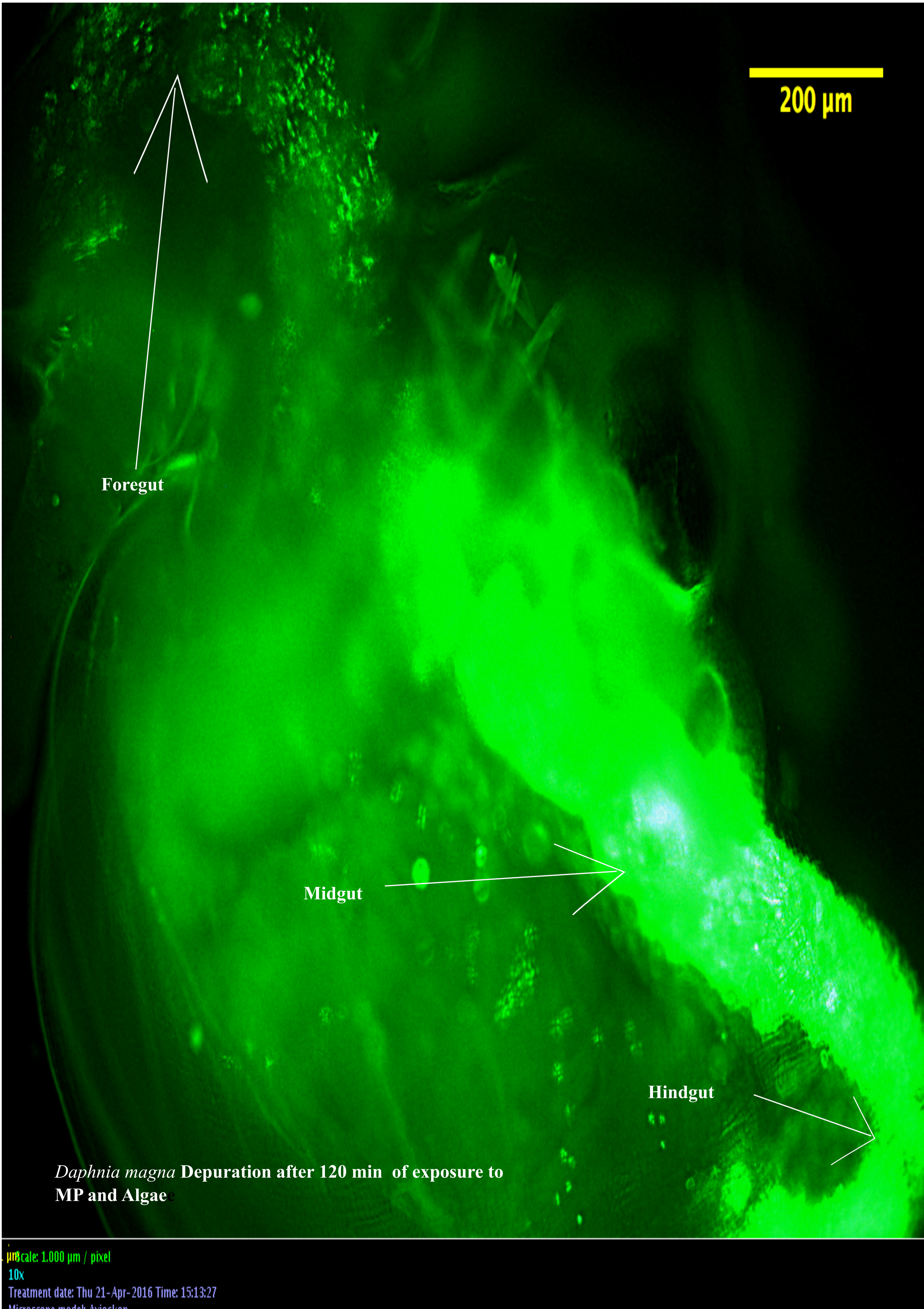

Daphnia magna **Depuration after 120 min of exposure to MP and Algae**
