# Supplementary material for: Impact of polystyrene microplastics on Daphnia magna mortality and reproduction in relation to food availability
Source: PeerJ. 2018 Apr 18;6:e4601. doi: 10.7717/peerj.4601 (PMC5911131; doi:10.7717/peerj.4601)
Supplement: Table S1 [file peerj-06-4601-s021.docx]

The average number of MP uptake ± standard error in treatments exposed to MP only over time.

| **Time /min** | **Average Number of microplastics** | **Standard error** |
| --- | --- | --- |
| 15 | 2572.4 | ±173.6 |
| 30 | 2229.1 | ±310.0 |
| 60 | 1884.9 | ±549.4 |
| 120 | 2806.9 | ±672.7 |
| 240 | 3349.6 | ±524.8 |
