# Supplementary material for: Impact of polystyrene microplastics on Daphnia magna mortality and reproduction in relation to food availability
Source: PeerJ. 2018 Apr 18;6:e4601. doi: 10.7717/peerj.4601 (PMC5911131; doi:10.7717/peerj.4601)
Supplement: Table S2 [file peerj-06-4601-s022.docx]

The average number of MP uptake ± standard error in treatments exposed to MP algae over time.

| **Time /min** | **Average Number of microplastics** | **Standard error** |
| --- | --- | --- |
| 15 | 1575.9 | ±360.7 |
| 30 | 1467.75 | ±171.8 |
| 60 | 849.75 | ±52.8 |
| 120 | 881.5 | ±41.4 |
| 240 | 757.05 | ±62.4 |
