# Supplementary material for: Impact of polystyrene microplastics on Daphnia magna mortality and reproduction in relation to food availability
Source: PeerJ. 2018 Apr 18;6:e4601. doi: 10.7717/peerj.4601 (PMC5911131; doi:10.7717/peerj.4601)
Supplement: Table S3 [file peerj-06-4601-s023.docx]

The average number of microplastics in the gut of *Daphnia magna* in treatments exposed to microplastics only after excretion in different times.

| **Time /min** | **Average Number of microplastics** | **Standard Error** |
| --- | --- | --- |
| 15 | 2163 | **±**74.6 |
| 30 | 2139.8 | **±**79.1 |
| 60 | 1738.1 | **±**58.3 |
| 120 | 1900.35 | **±**179.5 |
| 240 | 1514.1 | **±**135.2 |
