# Supplementary material for: Impact of polystyrene microplastics on Daphnia magna mortality and reproduction in relation to food availability
Source: PeerJ. 2018 Apr 18;6:e4601. doi: 10.7717/peerj.4601 (PMC5911131; doi:10.7717/peerj.4601)
Supplement: Table S4 [file peerj-06-4601-s024.docx]

The average number of microplastic in the gut of *Daphnia magna* in treatments exposed to microplastics and algae after excretion in different times.

| **Time /min** | **Average Number of microplastics** | **Standard Error** |
| --- | --- | --- |
| 15 | 393.9 | **±**19.4 |
| 30 | 393.9 | **±**26.3 |
| 60 | 393.9 | **±**26.3 |
| 120 | 332.2 | **±**7.7 |
| 240 | 339.9 | **±**52 |
